# Student and Physician Views of How the Dobbs Decision Affects Training and Practice Location Preferences: Cross-Sectional Questionnaire Study

**DOI:** 10.2196/55035

**Published:** 2025-01-07

**Authors:** Morgan S Levy, Simone A Bernstein, Sarah M McNeilly, Abigail Liberty, Shira Fishbach, Shikha Jain, Jessica A Gold, Vineet M Arora

**Affiliations:** 1 Department of Radiation Oncology University of Kentucky College of Medicine Lexington, KY United States; 2 Department of Psychiatry Washington University School of Medicine in St. Louis St. Louis, MO United States; 3 Albert Einstein College of Medicine New York, NY United States; 4 Department of Obstetrics and Gynecology Oregon Health and Science University Portland, OR United States; 5 Department of Obstetrics and Gynecology University of Michigan School of Medicine Ann Arbor, MI United States; 6 Department of Medicine University of Illinois at Chicago Chicago, IL United States; 7 Department of Medicine University of Chicago Pritzker School of Medicine Chicago, IL United States

**Keywords:** abortion, physician workforce, social media, reproductive health, medical education, abortion access, education, survey study, students, training, patient care, care, medical students, human rights, autonomy

## Abstract

**Background:**

By allowing for abortion bans and restrictions to take effect in the majority of US states, the 2022 *Dobbs v Jackson Women’s Health Organization* decision portends to have lasting impacts on patient care and the physician workforce. Notably, it is already beginning to impact practice location preferences of US health care workers, evidenced by declining application rates to residency programs in abortion-restrictive states since 2022. Yet, there remains a gap in the literature regarding why this trend exists.

**Objective:**

This study aims to describe what factors are driving the practice location preferences of medical students and physicians after the *Dobbs* decision.

**Methods:**

This study analyzes qualitative data from a web-based, cross-sectional study. In August 2022, a nonprobabilistic sample of physicians and medical students were surveyed on social media about the impact of overturning *Roe v Wade* on practice location preferences, which included the free-text question “Please share your thoughts about the overturning of *Roe v Wade* and how it will affect your decision about your (residency/job or fellowship) programs.” A total of 3 independent team members completed an inductive thematic analysis of 524 free responses, resolving differences by discussion.

**Results:**

Approximately 1 in 4 survey respondents also completed the free-response item (524/2063, 25.4%); a total of 219 were medical students, 129 were residents and fellows, and 176 were practicing physicians. Of them, approximately half (261/524, 50.5%) resided in states where abortion bans were in place or anticipated. Those who answered the free-response item were relatively more likely to hail from states with restrictive abortion bans (*P*<.001) compared to those who did not, with other demographic characteristics being largely similar between the groups. Inductive thematic analysis yielded 2 broad thematic categories: patient-related and workforce-related factors influencing practice decision preferences. The 3 most common themes overall were respondent concerns regarding their patient’s access to care (249/524, 47.5%), their desire not to practice or train in a state with abortion restrictions regardless of current residence (249/524, 47.5%), and their personal belief that abortion bans are human rights and/or body autonomy violation (197/524, 37.6%). Some respondents stated that the *Dobbs* decision would not impact their choice of practice location (41/524, 7.8%), and some supported it (35/594, 6.7%).

**Conclusions:**

This study shows that abortion restrictions are having an impact on the practice location preferences of the physician workforce due to both patient care and personal factors. It is important that state policy makers and others who are considering abortion restrictions also consider how to address these concerns of physicians and medical students, to avoid worsening geographic maldistribution of physicians and worsening access to care from physicians for their citizens.

## Introduction

The US landmark *Dobbs v Jackson Women’s Health Organization* decision allowed for widespread restrictions on abortion care, with 14 US states now enforcing total abortion bans and 27 more with bans based on gestational age [1,2]. These include Targeted Regulation of Abortion Providers (TRAP) laws that hamper and criminalize the practice of abortion [2].

While evidence affirms that abortion restrictions have deleterious effects on patient care and public health [3-6], it is important to understand that such policies also impact the health of physicians. A majority of physicians and medical students plan to build families during or after medical training, with thousands desiring pregnancy each year [7,8]. Many rely on infertility treatments, which abortion restrictions hamper [9]. Abortion restrictions, therefore, may deny a significant proportion of the physician workforce comprehensive family planning services, placing them at risk of forced birth [10]. Furthermore, they may also create moral injury among physicians from conflict between personal and professional morals, uncertainty regarding allowable practices, and fear of prosecution [11]. Those who provide abortion care may face increased stigma or even criminalization, depending on the state in which they train or practice. Those who are in restricted states and are not able to provide abortion care may struggle to navigate what is right for their patients versus what is legal, potentially worsening burnout and compassion fatigue [11,12].

Recent analysis from the American Association of Medical Colleges (AAMC) shows that fewer US MD seniors applied for residency positions in abortion-banned states versus nonban states in 2023 [13]. This includes a small but significant decline in the number of applications to obstetrics and gynecology residency programs in restrictive states in 2022 and 2023 [14]. To date, no study has described why physicians hold such preferences. Using an inductive analysis of free-response survey questions from our previous survey, this study aims to describe how state abortion restrictions may influence physicians’ and students’ decisions about where to live and practice.

## Methods

### Overview

We conducted a web-based, cross-sectional study for 2 weeks in August 2022. A nonprobabilistic sample of physicians (practicing physicians, fellows, and residents) and medical students were recruited from dedicated physician communities on social media (Twitter [rebranded as X in July 2023], Facebook, and Instagram [Meta Platforms]) through platforms like the American Medical Women’s Association and Inside The Match. All physicians and medical students in the United States were eligible to participate, including both those who practice or intend to practice in reproductive health care and those who do not. There was no minimum age for participation. Physicians completed a questionnaire about the impact of overturning *Roe v Wade* on practice location preferences [15]. Respondents reported demographic information and their location preferences for residency (medical students) or fellowship and jobs (physicians). No identifying information was collected.

This analysis focused on the study respondents’ stated practice location preferences. Quantitative data from this study were previously published [15]. Survey respondents were offered a free-response question, “Please share your thoughts about the overturning of *Roe v Wade* and how it will affect your decision about your (residency/job or fellowship) programs.” An inductive thematic analysis was used [10]. We consulted the Standards for Reporting Qualitative Research to report the study findings [16]. The free-response item was included to allow respondents to contextualize their practice location preferences [17]. The study team is comprised of a medical student pursuing obstetrics and gynecology (OBGYN; SMM), residents in radiation oncology (MSL) and OBGYN (SF), a fellow in Complex Family Planning (AL), and practicing physicians in psychiatry (SAB and JAG) and internal medicine (SJ and VMA). Some team members practice or are training in locations with abortion restrictions, and some practice in less restrictive locations. A total of 4 independent coders (MSL, SAB, SMM, and SF) coded responses until thematic saturation was reached (n=73 for medical students and n=102 for residents, fellows, and practicing physicians) and established the code book through consensus discussion. After establishing the code book, 2 authors coded all responses (n=524), and differences were resolved by discussion. Statistics were done in IBM SPSS (version 29), and group comparisons were calculated with chi-square testing. The CHERRIES checklist for the reporting of internet surveys guided the reporting of the study (Multimedia Appendix 1) [18].

### Ethical Considerations

The study was approved as exempt from review by the Institutional Review Board at the University of Chicago (IRB22-1066). Participants provided consent with the opportunity to opt out of the study and were not compensated for participation. Data were collected without identifiers and are only accessible to study team members.

## Results

### Demographics

Of the 2063 survey respondents, 524 (25.4%) completed the free-response item. Respondents consisted of medical students (n=219), residents and fellows (n=129), and practicing physicians (n=176). Most identified as cisgender women (391/524, 74.6%). The majority (453/524, 86.5%) of respondents were of reproductive age (less than age 44) and had no children (361/524, 68.9%). Approximately half (261/524, 50.5%) resided in states where abortion bans were in place or anticipated; half (256/524, 49.5%) resided in states where abortion remains legal [19]. Roughly a fifth (114/524, 21.8%) specialized in OBGYN, 13.2% (69/524) specialized in family medicine, and 65.1% (341/524) specialized in another field. The complete demographics of the sample who answered the free-response portion appear in Table 1.

Respondents who answered the free-response item were similar to those who did not by gender (*P*=.07), race (*P*=.13), or whether they intended to provide abortion care (*P*=.22). Respondents in states with restrictive abortion bans (50.5%) were more likely to respond (*P*<.001) compared with those in a state without restrictive abortion bans (41.7%).

Free-response rates suggest that these qualitative data appropriately represent the spectrum of views on abortion rights and access. Of the overall sample, 82.3% (1698/2063) indicated they would prefer to apply where abortion access is preserved; among them, 23.1% (393) answered the free-response item versus 76.9% (1305/2063) who did not (*P*<.001). However, of the 9.7% (200/2063) who did not prefer to apply where abortion access was preserved, 41.5% (83) provided a free response, while 58.5% (117) did not (*P*<.001). Of the 11.1% (229) who indicated that abortion restrictions do not impact their preferences, 32.8% (75) responded versus 67.2% (154) who did not (*P*<.001).

**Table 1:** Demographics of medical students (n=219), residents and fellows (n=129), and practicing physicians (n=176) who answered the free response item.

Characteristic			Total (n=524), n (%)		Medical students (n=219), n (%)^a^		Residents and fellows (n=129), n (%)		Practicing physicians (n=176), n (%)
**Gender^b^**									
	Woman	391 (74.6)		158 (72.1)		94 (72.9)		139 (79)	
	Man	109 (20.8)		48 (21.9)		30 (23.3)		31 (17.6)	
	Transgender and/or gender nonconforming	7 (1.4)		4 (1.9)		2 (1.6)		1 (0.6)	
	Prefer to describe	12 (2.3)		2 (0.9)		0 (0)		3 (1.7)	
	Prefer not to answer	43 (2.1)		7 (3.2)		3 (2.3)		2 (1.1)	
**Ethnicity^c^**									
	Hispanic	45 (8.6)		27 (12.3)		10 (7.8)		8 (4.5)	
	Not Hispanic	456 (87)		181 (82.6)		114 (88.4)		161 (91.5)	
	Prefer not to answer	23 (4.4)		11 (5)		5 (3.9)		7 (4)	
**Race^c^**									
	American Indian, Alaska Native, Native Hawaiian, or other Pacific Islander	2 (0.4)		2 (1)		0 (0)		0 (0)	
	Asian	49 (9.4)		18 (8.2)		9 (7)		22 (12.5)	
	Black, African American, or African	37 (7.1)		19 (8.7)		16 (12.4)		2 (1.1)	
	Multiracial^d^	27 (5.2)		13 (5.9)		6 (4.7)		8 (4.5)	
	White	361 (68.9)		140 (63.9)		91 (70.5)		130 (73.9)	
	Prefer to describe	15 (2.9)		8 (3.7)		1 (0.8)		6 (3.4)	
	Prefer not to answer	33 (6.3)		19 (8.7)		6 (4.7)		8 (4.5)	
**Sexual orientation**									
	Bisexual	51 (9.7)		30 (13.7)		8 (6.2)		13 (7.4)	
	Gay or lesbian	19 (3.6)		8 (3.7)		4 (3.1)		7 (4)	
	Heterosexual	404 (77.1)		157 (71.7)		106 (82.2)		141 (80.1)	
	Queer, pansexual, and/or questioning	21 (4)		7 (3.2)		6 (4.7)		8 (4.5)	
	Don’t know	3 (0.6)		3 (1.4)		0 (0)		0 (0)	
	Prefer to describe	6 (1.1)		4 (1.8)		0 (0)		2 (1.1)	
	Prefer not to answer	20 (3.8)		10 (4.6)		5 (3.9)		5 (2.8)	
**Age range^e^ (years)**									
	≤44	453 (86.5)		218 (99.5)		127 (98.4)		68 (38.6)	
	≥45	71 (13.5)		1 (0.5)		2 (1.6)		108 (61.4)	
**Relationship status**									
	Single	128 (24.4)		72 (32.9)		29 (22.5)		27 (15.3)	
	Partnered	125 (23.9)		89 (40.6)		29 (22.5)		7 (4)	
	Married	251 (47.9)		53 (24.2)		68 (52.7)		130 (73.9)	
	Widowed	2 (0.4)		0 (0)		0 (0)		2 (1.1)	
	Divorced	5 (1)		1 (0.5)		0 (0)		4 (2.3)	
	Other	5 (1)		1 (0.5)		1 (0.8)		3 (1.7)	
	Prefer not to answer	8 (1.5)		3 (1.4)		2 (1.6)		3 (1.7)	
**Children**									
	Yes	163 (31.1)		25 (11.4)		22 (17.1)		116 (65.9)	
	No	361 (68.9)		194 (88.6)		107 (82.9)		60 (34.1)	
**Respondent’s current state of residence, by anticipated abortion restriction^f^**									
	Ban or likely ban^g^	261 (50.5)		125 (58.4)		66 (51.2)		70 (40.2)	
	Legal^h^	256 (49.5)		89 (41.6)		63 (48.8)		104 (59.8)	
**Specialties**									
	Obstetrics and gynecology	114 (21.8)		51 (23.3)		28 (21.7)		35 (19.9)	
	Family medicine	69 (13.2)		30 (13.7)		17 (13.2)		22 (12.5)	
	All others	341 (65.1)		138 (63.0)		84 (65.1)		119 (67.6)	

^a^Includes US medical students (n=188) and international medical graduates applying to US residency programs (n=31).

^b^Nationally, medical students are 47.9% female and 52.9% male, residents and fellows are 46.8% female and 53% male, and practicing physicians are 35.9% female and 64.1% male [20].

^c^Nationally, medical students are 0.2% American Indian or Alaska Native, 54.6% White, 21.6% Asian, 6.2% Black or African American, 5.3% Hispanic, 8% multiple races, and 3.5% other. Nationally, residents and fellows are 0.11% American Indian or Alaska Native, 48.9% White, 26.6% Asian, 6% Black or African American, 9.2% Hispanic, 4% multiple races, and 3.1% other. Nationally, practicing physicians are 0.1% American Indian or Alaska Native, 63.9% White, 19.2% Asian, 3.6% Black or African American, 5.5% Hispanic, 2% multiple races, and 5.6% other [20,21].

^d^Respondents who selected more than one option are considered multiracial for the purpose of this study.

^e^Age 15-44 years is defined as reproductive age per the Centers for Disease Control and Prevention [22].

^f^Includes all 50 states, Puerto Rico, and the District of Columbia. Excludes the 7 respondents who indicated “other” on their location [19].

^g^Alabama, Arizona, Arkansas, Florida, Georgia, Idaho, Indiana, Iowa, Kentucky, Louisiana, Michigan, Mississippi, Missouri, Montana, North Carolina, North Dakota, Ohio, Oklahoma, South Carolina, South Dakota, Tennessee, Texas, Utah, West Virginia, Wisconsin, and Wyoming [19].

^h^Alaska, California, Colorado, Connecticut, Delaware, District of Columbia, Hawaii, Illinois, Kansas, Maine, Maryland, Massachusetts, Minnesota, Nebraska, Nevada, New Hampshire, New Jersey, New Mexico, New York, Oregon, Pennsylvania, Puerto Rico, Rhode Island, Vermont, Virginia, and Washington [19].

### Overview of the Inductive Analysis of Free-Response Survey Answers

There were 2 groups of themes and 2 stand-alone themes. One group described how practice location decisions impact patient care (Table 2), and the other captured workforce-related concerns (Table 3). The remaining themes included no impact and antiabortion sentiment.

**Table 2:** Patient factors influencing decisions about practice location emerging from the inductive analysis of the following: for students applying to residency, “Please share your thoughts about the overturning of Roe v Wade and how it will affect your residency application and ranking decisions below,” and for fellows and practicing physicians, “Please share your thoughts about the overturning of Roe v Wade and how it will affect your decision about your job or fellowship programs” among respondents (n=524).

Theme	Students (n=219), n (%)	Example quote	Physicians (n=305), n (%)	Example quote
Patient access to abortion (or reproductive care)	84 (38)	I’m horrified when I imagine taking care of a teenager who is being forced to carry out a pregnancy. I’m terrified of the burden of caring for a NICU filled with babies who were born despite having anomalies that make their short lives painful. I can only hope I’m not assaulted or become pregnant without the option to terminate.	165 (54)	I want to be able to support my patients to make good decisions about pregnancy. I need to be able to refer people if they need termination of pregnancy. It goes against my ethics to have to deprive someone of that option. I care foremost about my patients. If one of my patients died because she couldn’t get an abortion, I wouldn’t be able to live with myself.
Did not want politics to interfere with medical care decisions	45 (20)	I never want to be in a situation where I face disciplinary and/or legal consequences for reporting a patient who is miscarrying (spontaneous or induced), and with the current climate, I genuinely fear that we may be moving toward the criminalization of abortion in many places. That risk is not worth it to me when I could train in so many other places.	78 (25)	A politician is unable to grasp the grey areas of obstetric care and the heartbreaking scenarios we encounter. It is bad enough that hospital administrators police our obstetric practice; we do not need another non-medical person telling us how to practice evidence-based medicine.
Challenges of providing any reproductive care to patients with an abortion ban	41 (19)	I was previously set on Ob-Gyn, but I am now looking seriously at other fields because of the politics surrounding women's health care. I don't want to have to worry about legal repercussions for providing the best care to my patients. This has strongly turned me away from Ob-Gyn as a medical specialty.	65 (21)	As an abortion provider, I know that as much as I care about serving a population with unmet needs, the inevitability of burnout working in a place where abortion is severely limited would be too much.
Challenges of providing patient care that is not reproductive in nature	15 (7)	It will significantly impact the ability of every physician to provide care to their patients, regardless of their specialty, as many medical conditions are exacerbated by pregnancy status	47 (15)	I’m a dermatologist, and this affects our practice, too! We prescribe Accutane every day, and if a patient does become pregnant while on this drug due to contraceptive failure, we recommend termination. We prescribe lots of other teratogenic drugs as well for many different cutaneous diseases, especially methotrexate. I don’t know how I can practice in a state where pharmacists might refuse to fill MTX.

**Table 3:** Practice location decisions that are workforce-related emerging from the inductive analysis of the following: for students applying to residency, “Please share your thoughts about the overturning of Roe v Wade and how it will affect your residency application and ranking decisions below,” and for fellows and practicing physicians, “Please share your thoughts about the overturning of Roe v Wade and how it will affect your decision about your job or fellowship programs” among respondents (n=524).

Theme	Students (n=219), n (%)	Example quote	Physicians (n=305), n (%)	Example quote
Not choosing to practice or train in a state with abortion restrictions	77 (35)	This decision has heavily affected my residency application process. Amazing programs that I’ve highly considered are now at the bottom of my list.	172 (56)	Just finished residency and specifically did not even consider jobs in states that ban the full spectrum of reproductive healthcare or states that looked like they would consider a ban. Overturning of Roe made me basically have to ignore half the country during my search. But given the job market today, finding a position in a state that allows me to actually care for my patients wasn’t hard.
Personal belief that an abortion ban is a human rights/body autonomy violation	63 (29)	States that do not respect basic human rights are not places I wish to live or raise a family.	134 (44)	The overturning of Roe is the overturning of basic freedoms, the right to privacy, and bodily autonomy. It is the first step in overturning other rights. It is removing science from medicine. It threatens all doctors whether they provide abortion care or not. I’m likely to leave medicine, then practice in that environment and take those risks.
Access to training and education in abortion	43 (20)	I want to be part of a program where abortion training is easily accessible, and I will not have to go out of state to get this training. I also want to protect these rights for myself and my future patients.	15 (5)	One of my biggest decisions in choosing my state of residency was to allow me every opportunity to learn about women’s care at all levels. The overturning will prevent students and residents from reaching their full potential of learning care for women. It is truly unfortunate that men outside of the walls of understanding of medical knowledge think they have the authority to control not only women’s bodies but also the education of those to be able to treat women in emergency settings safely and holistically.
Personal or family access to abortion care or family building	36 (16)	I’m a guy, but what about my daughters in the future? What about a pregnancy complication with my wife? What about my patients? This is the problem when people claim moral high ground on the basis of their religion and are placed into positions of power; you end up with a sort of theocracy.	58 (19)	I was planning on looking for underserved community jobs in Idaho, but now that they have an early abortion ban, I will not be. I am actively trying to get pregnant and won’t risk my life to pursue a job.
Geographic ties to states with abortion restrictions limiting relocation	18 (8)	I attend medical school in my home state, which hasn't banned abortion as of yet but might do so in the future. If abortion is banned here, I'll likely still rank in-state programs due to the proximity of my family, but I will not rank out-of-state programs where abortion is banned.	38 (12)	Unfortunately, my answers are influenced by the fact that I live in a state with some of the most restrictive policies and have no ability to move. I cannot simply uproot my life to another state due to my feelings on abortion access. I work here, and my husband works here. My family is here. His family is here. The best I can do is to advocate for change, but I must remain in place as the primary breadwinner in my family.
Challenges recruiting to states with abortion restrictions	0 (0)		18 (6)	I’m a program director and am concerned about how this will affect recruiting talented and eager physicians to our state. Our patients already have difficulty accessing the medical system, so if this decision leads to physicians leaving the state, it will only amplify disparities.

### Patient Factors Influencing Decisions About Practice Location

#### Patient Access to Abortion or Full-Spectrum Reproductive Care

Many physicians and medical student respondents want patients to have access to safe and legal abortion. Respondents specifically highlighted concerns that adolescents, underrepresented minority groups, people in rural communities, and lower-income patients would increasingly face challenges in finding abortion providers, exacerbating health disparities (Table 2).

Physicians also noted that restrictions interfere with their ability to provide or refer patients for abortion care. For example, one stated, “I won’t practice in a state that limits my ability to provide or refer my patients for care that is safe and necessary for their health and well-being.”

#### Challenges of Providing Reproductive Care to Patients During an Abortion Ban

Reproductive health care providers anticipate moral distress if they are unable to provide abortion care in circumstances like lethal fetal anomalies or pregnancies resulting from rape or incest. An OBGYN physician explained, “Abortion care and prenatal care go hand in hand. This is a field with a lot of gray areas, and elimination of options will harm those who can get pregnant.” Many physicians feared legal repercussions and were disappointed by a perceived lack of institutional support for evidence-based health care.

#### Do Not Want Politics to Interfere With Medical Care Decisions

Some respondents expressed concern that lawmakers are interfering with medical care. Others emphasized the role physicians play in advocacy and supporting elected officials in favor of essential reproductive health care. A participant stated, “The government should have no standing in a medical decision between physician and patient.”

#### Challenges of Providing Patient Care That Is Not Reproductive in Nature

Physicians across various fields were concerned that abortion restrictions would adversely impact their clinical practice. For example, a pediatrician noted, “Working with fetal cardiac patients, it is imperative that my patients have access to abortion services if that’s the choice they make that’s best for their families.” In addition, an oncologist worried about restrictions on chemotherapy regimens, a dermatologist had questions about prescribing common medications (like Accutane) that are teratogenic, and a rheumatologist had concerns about prescribing methotrexate.

### Workforce-Related Practice Location Decisions

#### Choosing Not to Practice or Train in a State With Abortion Restrictions

Many respondents living in states with abortion protections stated that they would be unwilling to move to a state with abortion restrictions (Table 3). Others living in restrictive states intend to move or preferentially apply to and rank training programs in states without abortion bans. Trainees described how these decisions compound their stress regarding the highly competitive match process. Some still felt pressured to apply everywhere, regardless of their personal preferences, stating, “Residency is already so competitive, so unfortunately, I feel like I have to apply everywhere, but I would definitely preferentially rank somewhere that I would have access to abortion care and that my patients would as well.”

#### Challenges Recruiting to States With Abortion Restrictions

Some residency and fellowship program directors and administrative leadership in states with restrictive abortion laws are concerned about recruiting and retaining residents, fellows, and faculty. Many foresee the reluctance of trainees and faculty to work in restrictive states. A program leader said, “I am an APD at an academic medical center in the Midwest. I have already been told by two residents that they had planned to stay in the state to practice but are now leaving solely because of the lack of reproductive rights in our state. I fear we will rapidly lose amazing physicians.”

#### Personal Belief That Abortion Restrictions Violate Human Rights and/or Bodily Autonomy

A substantial portion of respondents described the overturning of *Roe v Wade* as a human rights violation and criticized its negative impact on patients’ bodily autonomy. Others discussed the potential moral injury from practicing in a state whose laws and policies prevent clinicians from providing evidence-based medical care.

Respondents connected states’ abortion policies to their overarching sociopolitical climates, noting that bans and restrictions may portend other harmful (eg, racist, homophobic, transphobic) policies. A medical student said, “Extremely cautious about applying to these states who have denied abortion care. Not only because of abortion care but also because these states are notoriously anti-LGBTQ+ and hold racist values. I do not want to live and work and raise a family in that environment, where I am not respected and have less human rights than others.”

#### Access to Abortion Training and Education

Students applying to OBGYN and family medicine expressed that their application decisions would be shaped by access to proper training in abortion care. Applicants to residency and fellowship recognize that selecting programs in abortion-restricted states may limit access to adequate training. Multiple students noted that they intend to inquire about abortion training during the residency application process.

Some recognized that trainees in abortion-restricted states could seek abortion training out-of-state. For example, a respondent said, “I plan to first rank programs in states with full spectrum reproductive health access, followed by programs that are intentional about providing training for their residents with full support (financial, housing, etc) to leave the state for abortion training.” However, current trainees also discussed challenges in obtaining abortion training, including professional, administrative, and financial barriers.

#### Geographic Ties to States With Abortion Restrictions Limiting Relocation

Some noted that geographic relocation is a privilege not afforded to everyone equally. The decision to move is often influenced by distance to a support network, job benefits for the respondent or their spouse, housing, and childcare. Such geographic ties discourage or prevent many medical students and physicians from leaving their state of residence despite their personal or professional opposition to abortion restrictions.

Some said they understand the risks of staying in a state with abortion restrictions. If necessary, they would travel out of state to receive an abortion, again recognizing their mobility is a privilege. A respondent said, “I definitely would prefer to be in a state that maintains access to abortion. Unfortunately, those are not states where my family lives, and I am grateful that I have enough privilege if I needed an abortion, I could leave the state.”

#### Personal or Family Access to Abortion Care or Family Building

Respondents were concerned about practicing in a location that limits their options for receiving comprehensive reproductive health care. Multiple respondents highlighted that they did not want to be forced to carry a pregnancy if they could not get an abortion, especially during training. Others specifically cited medical conditions that would make pregnancy physically challenging and even contraindicated as a reason to ensure they had access to abortion care. A respondent said, “I am a medical student with chronic conditions that make pregnancy life-threatening for me. Although I am on contraceptives, nothing is 100%, and I want to be able to protect my life and well-being in case I do accidentally get pregnant.”

In addition, physicians with infertility undergoing in vitro fertilization noted that practicing in a state where life is defined as beginning at fertilization would make family building significantly more challenging. Commonly, respondents stated they were concerned about care for themselves, their children, or their partners, underscoring the importance of recognizing that physicians, too, need access to care.

### Additional Themes

#### No Impact

Few medical students and physicians stated that the *Dobbs* decision would not impact their choice of practice location (Table 4). Some indicated that the residency and fellowship match were too competitive to make decisions based on abortion legislation. For example, those who apply to every program in their field may end up applying to programs in states with abortion restrictions to increase their likelihood of matching.

**Table 4:** Practice location decisions that are workforce-related emerging from the inductive analysis of the following: for students applying to residency, “Please share your thoughts about the overturning of Roe v Wade and how it will affect your residency application and ranking decisions below,” and for fellows and practicing physicians, “Please share your thoughts about the overturning of Roe v Wade and how it will affect your decision about your job or fellowship programs” among respondents (n=524).

Theme	Students (n=219), n (%)	Example quote	Physicians (n=305), n (%)	Example quote
No impact	30 (14)	Matching and getting into a program is challenging enough considering the various factors at play; this decision will not be part of deciding which states or programs I end up applying to.	11 (4)	It will have zero impact on my decisions regarding jobs/fellowships.
Expressed support for overturning *Roe v Wade*	17 (8)	The overturning of Roe v. Wade is long overdue. It was not right in the first place, as the Supreme Court made clear in its ruling. Babies deserve to live inside and outside the womb.	18 (6)	I am supportive of the overturn and believe it will be better for our patients and medical care to ban an inhumane practice like abortion. Human lives in the womb deserve protection just like all of our other patients at any age and ability.

#### Expressed Antiabortion Sentiment and/or Support for Overturning Roe v Wade

Physicians and medical students who expressed antiabortion (“pro-life”) views supported the Supreme Court decision (Table 4). Multiple respondents noted that they would purposefully seek out practice environments where abortion restrictions existed. Reasons for this include not supporting abortion care for any indication, stating that they do not view abortion as health care, a desire to “preserve life,” and a desire to “protect the unborn.” Multiple respondents discussed that abortion is an issue that should be legislated at the state level.

## Discussion

### Principal Findings

Our study shows that abortion restrictions will have a substantial impact on the physician workforce in patient care and practice location decisions. The 3 most common themes were patient access to care, not choosing to practice or train in a state with abortion restrictions, and personal belief that an abortion ban is a human rights/body autonomy violation. This study enhances emerging literature about the impacts of abortion restrictions on the physician workforce, including physicians and medical students at all levels of training across all 50 states within both reproductive and nonreproductive health fields.

Respondents shared concerns that abortion restrictions will negatively impact their ability to provide high-quality, comprehensive reproductive care. This was evident among trainees who provide abortion care, like OBGYN residents, who expressed concerns about new or worsening barriers to obtaining foundational skills like first-trimester uterine aspiration at their primary institution [23,24]. OBGYN trainees also cited multiple barriers to obtaining foundational abortion care skills at their primary institutions. Some programs have created away rotation opportunities for residents unable to obtain comprehensive abortion training at their own institutions [25]. However, there are many barriers to these programs, including obtaining state-based medical licenses, getting funding and organizational affiliations in place, and disruptions to families when living in another state [25].

Even within nonreproductive health care fields, respondents shared concerns about the downstream effects of abortion restrictions on clinical training and practice. In the 2 weeks following the *Dobbs* decision, only 38.5% of a list of 187 societies across a wide variety of specialties had made a statement about the decision [26]. Respondents from specialties that do not provide abortion care noted concern for restricted use of potentially abortifacient or teratogenic medications and worsening health among patients whose physical or mental health will be adversely impacted by restrictions.

Physicians and medical students also worried that abortion restrictions would deleteriously affect their personal health and well-being. Restrictions hold significant health implications for reproductive-age women, a large and growing demographic of the physician workforce [27]. Recent studies have reported that abortion is common among physicians, affirming that physicians, too, need safe, legal access to abortion [28]. This study informs future medical education and occupational health research by elevating trainees’ and employees’ concerns. As highlighted by the medical student responses on geographic ties and competitiveness of the match process, it is critical to recognize the multifactorial decision-making involved in where to complete residency training. While before the *Dobbs* decision, telehealth may have been able to bridge the gaps in access to abortion care, this is less likely to be possible in the current landscape [29]. Medical schools and hospitals, especially those in restrictive states, must recognize this and prepare to navigate the adverse health, financial, and legal repercussions their employees may face. Otherwise, disparate abortion access may increase health disparities within the physician workforce and threaten its diversity and resiliency [30].

If medical students do not want to practice in states with abortion restrictions in place, it is less likely that they will establish their practice in those locations. In 2022, 55.2% of those completing training established their practice in the same state where they completed residency [31]. The lack of physicians who are willing to practice in states with abortion restrictions can further poor health outcomes in maternity care deserts [32,33]. Idaho is a notable example, where 41% of OBGYN physicians consider leaving and cite restrictive abortion laws as a motivation [34,35]. Idaho has the lowest rate of physicians per 100,000 people in the entire country [34,35].

Some physicians stated that abortion restrictions would not impact them or that they support them. Notably, a subset of “no impact” responders shared that the scarcity of available positions, particularly within highly competitive specialties and for historically marginalized applicants, outweighs their personal opposition to abortion restrictions. Others acknowledged the futility of setting preferences since the match is ultimately complex and multifactorial.

### Limitations

This study may be limited by self-selection bias, given its recruitment of medical students and practicing physicians on social media. Of the respondents who did not prefer to apply where abortion access was preserved, a substantial number (41.5%) provided a free response, indicating that we had a spectrum of views on abortion rights. Furthermore, this sample is focused on physicians and does not represent other health care workforce members who are likely also impacted by abortion restrictions.

### Conclusion

The findings of this study captured responses to abortion restrictions before the 2023 Match cycle and provided context to the recent AAMC data showing that residency applications disproportionately decreased in restrictive states [13]. Narrative responses bolster our original quantitative data, affirming that access to full-spectrum reproductive health care was highly valued personally and professionally by most physicians [15].

This study shows that abortion restrictions are having an impact on the practice location preferences of the physician workforce due to both patient care and personal factors. It is important that state policy makers and others who are considering abortion restrictions also consider how to address these concerns of physicians and medical students, to avoid worsening geographic maldistribution of physicians and worsening access to care from physicians for their citizens.

## Appendix

Multimedia Appendix 1 The Checklist for Reporting Results of Internet E-Surveys (CHERRIES).

## Abbreviations

AAMC: American Association of Medical Colleges
OBGYN: obstetrics and gynecology
TRAP: Targeted Regulation of Abortion Providers

## Data Availability

The datasets generated during and/or analyzed during this study are not publicly to protect the anonymity of participants due to the sensitive subject matter of this manuscript but are available from the corresponding author on reasonable request.
