# Supplementary material for: Student and Physician Views of How the Dobbs Decision Affects Training and Practice Location Preferences: Cross-Sectional Questionnaire Study
Source: Interact J Med Res. 2025 Jan 7;14:e55035. doi: 10.2196/55035 (PMC11751647; doi:10.2196/55035)
Supplement: Multimedia Appendix 1 [file ijmr_v14i1e55035_app1.pdf]

Checklist for Reporting Results of Internet E-Surveys (CHERRIES)

This checklist has been adapted from the original for Obstetrics & Gynecology. Source: Eysenbach G. Improving the quality of Web surveys: the Checklist for Reporting Results of Internet E-Surveys (CHERRIES). J Med Internet Res. 2004 Sep 29;6(3):e34 [erratum in J Med Internet Res. 2012; 14(1): e8.]. Article available at http://dx.doi.org/10.2196/jmir.6.3.e34; erratum available at http://dx.doi.org/10.2196/jmir.2042. Copyright ©Gunther Eysenbach. Originally published in the Journal of Medical Internet Research (http://www.jmir.org), 29.9.2004 and 04.01.2012. This is an open-access article distributed under the terms of the Creative Commons Attribution License (http://creativecommons.org/licenses/by/2.0/), which permits unrestricted use, distribution, and reproduction in any medium, provided the original work, first published in the Journal of Medical Internet Research, is properly cited. The complete bibliographic information, a link to the original publication on http://www.jmir.org/, as well as this copyright and license information must be included.

Checklist Item	***Explanation***	***Description***	***Page Number***
Describe survey design	Describe target population, sample frame. Is the sample a convenience sample? (In "open" surveys this is most likely.)	The target population is physicians and medical students. The sample is a convenience sample.	6
IRB approval	Mention whether the study has been approved by an IRB.	The study was approved by the Institutional Review Board at the University of Chicago.	6
Informed consent	Describe the informed consent process. Where were the participants told the length of time of the survey, which data were stored and where and for how long, who the investigator was, and the purpose of the study?	For screening the participants were asked if they were a physician or medical student. Participants were informed that the study would take 5 minutes, that data would be stored in a secure location, names and contact information for the principal investigators and IRB, and study purpose.	N/A
Data protection	If any personal information was collected or stored, describe what mechanisms were used to protect unauthorized access.	Data was not collected with identifiers; however, data was stored in a secure location authorized by the IRB to protect participants.	N/A

Development and testing	State how the survey was developed, including whether the usability and technical functionality of the electronic questionnaire had been tested before fielding the questionnaire.	The survey was developed by the investigators on Qualtrics, and was tested by people not on the study team (whose data were cleared and not included in the analysis) for usability and technical functionality.	N/A
Open survey versus closed survey	An "open survey" is a survey open for each visitor of a site, while a closed survey is only open to a sample which the investigator knows (password-protected survey).	The survey was open to all who viewed the posts on social media.	N/A
Contact mode	Indicate whether or not the initial contact with the potential participants was made on the Internet. (Investigators may also send out questionnaires by mail and allow for Web-based data entry.)	The initial contact with participants was made on the internet via social media posts.	6
Advertising the survey	How/where was the survey announced or advertised? Some examples are offline media (newspapers), or online (mailing lists – If yes, which ones?) or banner ads (Where were these banner ads posted and what did they look like?). It is important to know the wording of the announcement as it will heavily influence who chooses to participate. Ideally the survey announcement should be published as an appendix.	The survey was advertised on social media (Facebook, Instagram, Twitter) from physician communities on social media.<br><br>A sample advertisement for one social media network appears in Appendix 1.	N/A
Web/E-mail	State the type of e-survey (eg, one posted on a Web site, or one sent out through e-mail). If it is an e-mail survey, were the responses entered manually into a database, or was there an	The survey was conducted on Qualtrics, which automatically compiles responses into a database.	N/A

	automatic method for capturing responses?		
Context	Describe the Web site (for mailing list/newsgroup) in which the survey was posted. What is the Web site about, who is visiting it, what are visitors normally looking for? Discuss to what degree the content of the Web site could pre-select the sample or influence the results. For example, a survey about vaccination on a anti-immunization Web site will have different results from a Web survey conducted on a government Web site	N/A - Study was posted on social media.	N/A
Mandatory/ voluntary	Was it a mandatory survey to be filled in by every visitor who wanted to enter the Web site, or was it a voluntary survey?	The study was voluntary.	N/A
Incentives	Were any incentives offered (eg, monetary, prizes, or non-monetary incentives such as an offer to provide the survey results)?	No incentives were offered to participants.	N/A
Time/Date	In what timeframe were the data collected?	The data was collected from August 12-23, 2022.	6
Randomization of items or questionnaires	To prevent biases items can be randomized or alternated.	Items were not randomized because the survey logic was built upon previous responses. The free response was the last item.	N/A

Adaptive questioning	Use adaptive questioning (certain items, or only conditionally displayed based on responses to other items) to reduce number and complexity of the questions.	Survey logic was included to ensure participants were only asked questions relevant to their level of medical practice.	N/A
Number of Items	What was the number of questionnaire items per page? The number of items is an important factor for the completion rate.	The number of items per page varied based on the length of the block of questions, no page contained more than 9 questions.	N/A
Number of screens (pages)	Over how many pages was the questionnaire distributed? The number of items is an important factor for the completion rate.	The questionnaire was distributed over 3 pages.	N/A
Completeness check	It is technically possible to do consistency or completeness checks before the questionnaire is submitted. Was this done, and if "yes", how (usually JAVAScript)? An alternative is to check for completeness after the questionnaire has been submitted (and highlight mandatory items). If this has been done, it should be reported. All items should provide a non-response option such as "not applicable" or "rather not say", and selection of one response option should be enforced.	This was not performed because participants were not required to answer this short-answer question in the survey due to the personal nature of the subject.	N/A
Review step	State whether respondents were able to review and change their answers (eg, through a Back button or a Review step which displays a summary of the responses and asks the respondents if they are correct).	Participants were not able to review and change answers.	N/A

Unique site visitor	If you provide view rates or participation rates, you need to define how you determined a unique visitor. There are different techniques available, based on IP addresses or cookies or both.	N/A	N/A
View rate (Ratio of unique survey visitors/unique site visitors)	Requires counting unique visitors to the first page of the survey, divided by the number of unique site visitors (not page views!). It is not unusual to have view rates of less than 0.1 % if the survey is voluntary.	N/A	N/A
Participation rate (Ratio of unique visitors who agreed to participate/unique first survey page visitors)	Count the unique number of people who filled in the first survey page (or agreed to participate, for example by checking a checkbox), divided by visitors who visit the first page of the survey (or the informed consents page, if present). This can also be called "recruitment" rate.	93% recruitment rate (2334/2522)	N/A
Completion rate (Ratio of users who finished the survey/users who agreed to participate)	The number of people submitting the last questionnaire page, divided by the number of people who agreed to participate (or submitted the first survey page). This is only relevant if there is a separate "informed consent" page or if the survey goes over several pages. This is a measure for attrition. Note that "completion" can involve leaving questionnaire items blank. This is not a measure for how completely questionnaires were filled in. (If you need a	81% completion rate (2036/2522)<br><br>This does not include 8 participants who were excluded because they were detected as likely to be bots by Qualtrics.	N/A

	measure for this, use the word "completeness rate".)		
Cookies used	Indicate whether cookies were used to assign a unique user identifier to each client computer. If so, mention the page on which the cookie was set and read, and how long the cookie was valid. Were duplicate entries avoided by preventing users access to the survey twice; or were duplicate database entries having the same user ID eliminated before analysis? In the latter case, which entries were kept for analysis (eg, the first entry or the most recent)?	N/A	N/A
IP check	Indicate whether the IP address of the client computer was used to identify potential duplicate entries from the same user. If so, mention the period of time for which no two entries from the same IP address were allowed (eg, 24 hours). Were duplicate entries avoided by preventing users with the same IP address access to the survey twice; or were duplicate database entries having the same IP address within a given period of time eliminated before	The survey was distributed through an anonymous link through Qualtrics, which collects the IP address and location of participants. The survey was set to not allow duplicate responses, this determination was made based on the IP address and location through the link.	N/A

	analysis? If the latter, which entries were kept for analysis (eg, the first entry or the most recent)?		
Log file analysis	Indicate whether other techniques to analyze the log file for identification of multiple entries were used. If so, please describe.	Not performed	N/A
Registration	In “closed” (non-open) surveys, users need to login first and it is easier to prevent duplicate entries from the same user. Describe how this was done. For example, was the survey never displayed a second time once the user had filled it in, or was the username stored together with the survey results and later eliminated? If the latter, which entries were kept for analysis (eg, the first entry or the most recent)?	N/A	N/A
Handling of incomplete questionnaires	Were only completed questionnaires analyzed? Were questionnaires which terminated early (where, for example, users did not go through all questionnaire pages) also analyzed?	Only completed questionnaires were analyzed. 524/2036 complete responses answered the free response item and were included in the inductive analysis.	N/A

Questionnaires submitted with an atypical timestamp	Some investigators may measure the time people needed to fill in a questionnaire and exclude questionnaires that were submitted too soon. Specify the timeframe that was used as a cut-off point, and describe how this point was determined.	Not performed	N/A
Statistical correction	Indicate whether any methods such as weighting of items or propensity scores have been used to adjust for the non-representative sample; if so, please describe the methods.	Not performed	N/A

Appendix 1: Advertisement for Social Media

Twitter:
📣 ALL doctors, residents/fellows, & med students

How does overturning Roe affect your decisions on where to work or train?

Take the short PRACTICE (Post Roe: Ascertaining Choices for Training and Career Expectations) Survey

https://tinyurl.com/PracticeStudySurvey

Graphic for Twitter

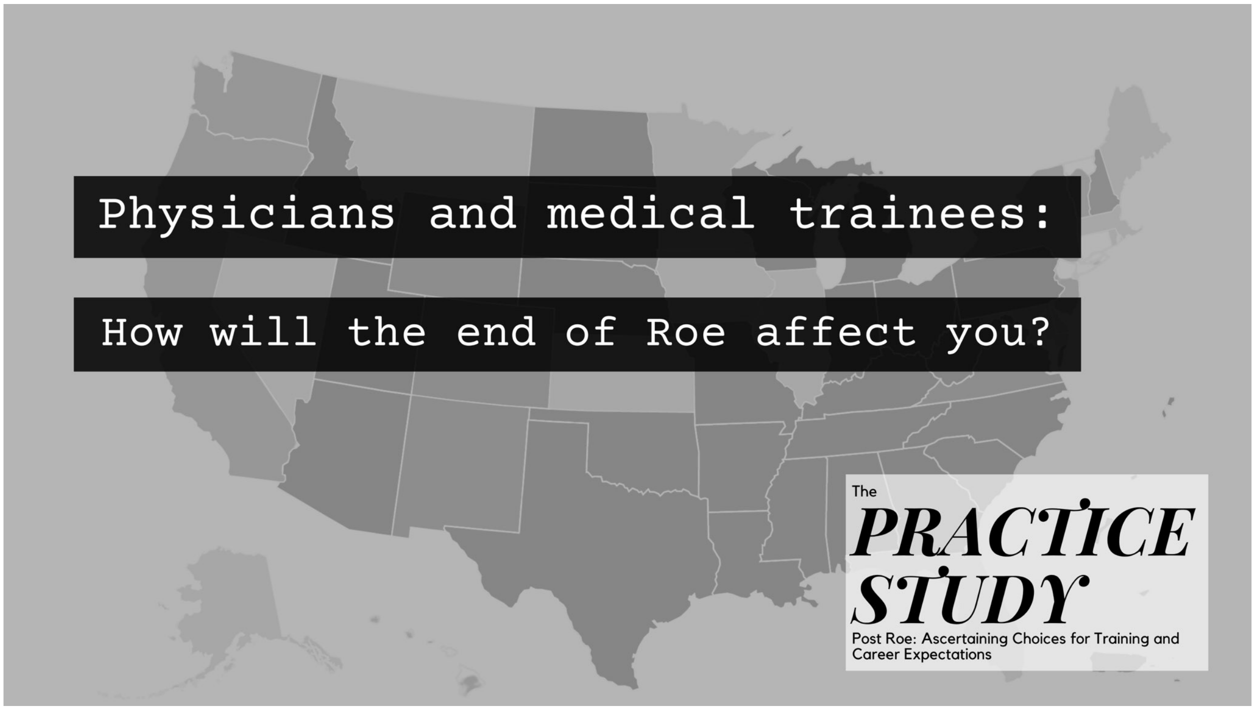

Physicians and medical trainees:
How will the end of Roe affect you?
The
PRACTICE
STUDY
Post Roe: Ascertaining Choices for Training and Career Expectations
